# Optimisation Models for Pathway Activity Inference in Cancer

**DOI:** 10.3390/cancers15061787

**Published:** 2023-03-15

**Authors:** Yongnan Chen, Songsong Liu, Lazaros G. Papageorgiou, Konstantinos Theofilatos, Sophia Tsoka

**Affiliations:** 1 Department of Informatics, Faculty of Natural, Mathematical and Engineering Sciences, King’s College London, Bush House, London WC2B 4BG, UK; 2 School of Management, Harbin Institute of Technology, Harbin 150001, China; 3 The Sargent Centre for Process Systems Engineering, Department of Chemical Engineering, University College London, Torrington Place, London WC1E 7JE, UK; 4 King’s College London British Heart Foundation Centre, School of Cardiovascular and Metabolic Medicine and Sciences, London SE1 7EH, UK

**Keywords:** pathway activity, RNA sequencing, optimisation, breast cancer, colorectal cancer

## Abstract

**Simple Summary:**

Subtype classification and prognostic prediction are key research targets in complex diseases such as cancer. In this work, an optimisation model was designed to infer the activity of biological pathways from gene expression values. The optimisation model enables the pathway activity values to separate the sample subtypes to the greatest extent, thereby improving sample classification accuracy. The proposed model was evaluated on cancer molecular subtype classification, robustness to noisy data and survival prediction, and allowed the identification of disease-important genes and pathways.

**Abstract:**

Background: With advances in high-throughput technologies, there has been an enormous increase in data related to profiling the activity of molecules in disease. While such data provide more comprehensive information on cellular actions, their large volume and complexity pose difficulty in accurate classification of disease phenotypes. Therefore, novel modelling methods that can improve accuracy while offering interpretable means of analysis are required. Biological pathways can be used to incorporate a priori knowledge of biological interactions to decrease data dimensionality and increase the biological interpretability of machine learning models. Methodology: A mathematical optimisation model is proposed for pathway activity inference towards precise disease phenotype prediction and is applied to RNA-Seq datasets. The model is based on mixed-integer linear programming (MILP) mathematical optimisation principles and infers pathway activity as the linear combination of pathway member gene expression, multiplying expression values with model-determined gene weights that are optimised to maximise discrimination of phenotype classes and minimise incorrect sample allocation. Results: The model is evaluated on the transcriptome of breast and colorectal cancer, and exhibits solution results of good optimality as well as good prediction performance on related cancer subtypes. Two baseline pathway activity inference methods and three advanced methods are used for comparison. Sample prediction accuracy, robustness against noise expression data, and survival analysis suggest competitive prediction performance of our model while providing interpretability and insight on key pathways and genes. Overall, our work demonstrates that the flexible nature of mathematical programming lends itself well to developing efficient computational strategies for pathway activity inference and disease subtype prediction.

## 1. Introduction

Deriving molecular signatures is essential for the diagnosis and prognosis of complex multi-factorial diseases such as cancers [1,2,3]. Gene signature identification methods that are associated with high-throughput technologies, such as Differentially Expressed Genes (DEGs) [4], have been widely used for this purpose and have been shown to outperform the traditional histopathological features [5,6]. However, the inherent “large-p, small-n” nature of high-throughput genomic data, where the amount of observations (n) is orders of magnitude smaller than the number of observable features (p), renders the identification of reliable and reproducible gene markers for heterogeneous diseases difficult [7,8]. Because the development of complex diseases is related to the disorder and perturbation of multiple genes, recent work has sought to incorporate a priori knowledge of gene sets to the disease classification problem in order to specify stable biological signatures [9,10,11,12,13].

Biological pathways are gene sets that reflect series of actions among molecules, encapsulating specific functional processes in cells and tissues such as signaling events or metabolic conversions. Determining which pathways are implicated in disease may enhance personalised strategies for diagnosis, treatment, and prevention of disease. A series of methods have been proposed to address this problem by constructing a new composite feature, called Pathway Activity (PA) [14], to transform gene-level data to pathway-level data. However, robustness and biological interpretability are challenging problems for existing pathway inference methods in trying to reduce dimensionality from a few thousands of genes in the entire genome to a few hundreds of pathways.

Popular baseline pathway activity inference methods in the literature include averaged gene expression [14], z-score [15], PCA [16,17,18,19], and the Bayes method [20]. These methods use statistical strategies or basic dimensionality reduction approaches to maintain a low level of data reconstruction errors. However, without biological insights (i.e., relationships or importance of pathway member genes) embedded in the inference procedure, the PA value differences between sample cohorts are difficult to explain. More advanced methods have been introduced for pathway activity inference. Ranking of pathway constituent genes according to their discriminative power and identifying a small set of highly differentially expressed genes has been reported [21]. Another method (Pathifier [19]) uses a principal curve generated from control samples to calculate the pathway deregulation score of disease samples. More recently, PASL has been proposed [12], with the aim of efficiently balancing biological interpretability and classification performance. While these advanced methods place greater emphasis on interpretability most of them remain statistical approaches, i.e., they rely on capturing the variance between sample cohorts. Therefore, their performance is highly dependent on the quality and volume of the input data. Moreover, most of these advanced methods are either applied to binary classification or are limited to a few signaling pathways [20,22], while the classification of multiple phenotypes remains challenging.

We have reported a supervised mathematical programming optimisation model, called DIGS (DIfferential Gene Signatures), which infers pathway activity as a weighted linear combination of pathway constituent genes [23,24]; it is one-of-a-kind among pathway activity inference methods, as it is based on optimisation principles. In DIGS, weights of features are optimised to maximise the discriminative power of the inferred pathway activity, in contrast to other methods where gene weights are set using prior knowledge (usually assuming averaged gene weights [14,25,26]). Furthermore, the supervised nature of DIGS is better suited to disease classification tasks than unsupervised methods, where pathway activity merely summarises the variance in molecular data. Lastly, the model is applicable to multi-class disease classification problems, and has been demonstrated to outperform other PA inferencing methods in classification tasks [23]. Overall, benefiting from its mathematical optimisation nature, DIGS ensures classification accuracy, while gene weights determined by the objective function provide DIGS with dual biological interpretability, deriving important pathways as well as important genes.

In this work, we aim to reduce the computational complexity of our previous mode; to this end, we propose a novel pathway activity optimisation model which avoids a large number of binary decision variables, converging more easily to optimality. In addition, we introduce a new way of evaluating the proposed method which does not rely on external Machine Learning classifiers, and can instead conduct disease phenotype prediction directly from the outputs of the new proposed model. Using RNA-Seq datasets of colorectal cancer and breast cancer from the Cancer Genome Atlas (TCGA) database, we show that the new optimisation model proposed here improves classification accuracy on multi-class problems compared to both the original model and to other pathway activity inference methods in the literature.

## 2. Materials and Methods

### 2.1. Data Preparation

Raw RNA-Seq count data were downloaded from the Cancer Genome Atlas (TCGA) database [27] along with corresponding clinical information. Two publicly available TCGA datasets, BRCA (for breast cancer samples) and COAD (for colorectal caner samples), were obtained [19,28,29,30]. Breast cancer is the most common malignancy in women globally [31], and colorectal cancer (CRC) is among the most lethal and prevalent malignancies worldwide [32]. Read count data were normalised by upper quartile FPKM (FPKM-UQ) [33] to obtain the gene expression profiles. Then, genes with very high missingness (over 30% zero expression values across the sample cohort) were removed. Table 1 shows the details of the TCGA datasets.

The COAD dataset consists of 521 samples in total, of which 480 are tumour tissue and 41 normal. For molecular subtypes, 85, 165, 58, and 120 samples belong to CMS1, CMS2, CMS3, and CMS4 respectively [34]. For BRCA, the total number of samples is 1211, with 1091 being tumour tissue samples and 120 being normal. PAM50 [35] was used to determine the molecular subtypes, and was retrieved using the TCGAbiolinks package [36]), resulting in 579 samples in Luminal A, 217 in Luminal B, 191 Basal samples, 82 Her2 samples, and 22 normal-like samples. The normal-like subtype accounts for about 5–10% of all breast carcinomas. These are poorly characterized, and have been grouped into the classification of intrinsic subtypes with fibroadenomas and normal breast samples [37,38].

Biological pathways were retrieved through the Kyoto Encyclopedia of Genes and Genomes (KEGG) API [39,40]. In total, 279 Homo Sapiens pathways were used in this study, with the number of genes in these pathways being 6761.

### 2.2. A Novel Optimisation-Based Pathway Activity Inference Model

**Figure 1:**
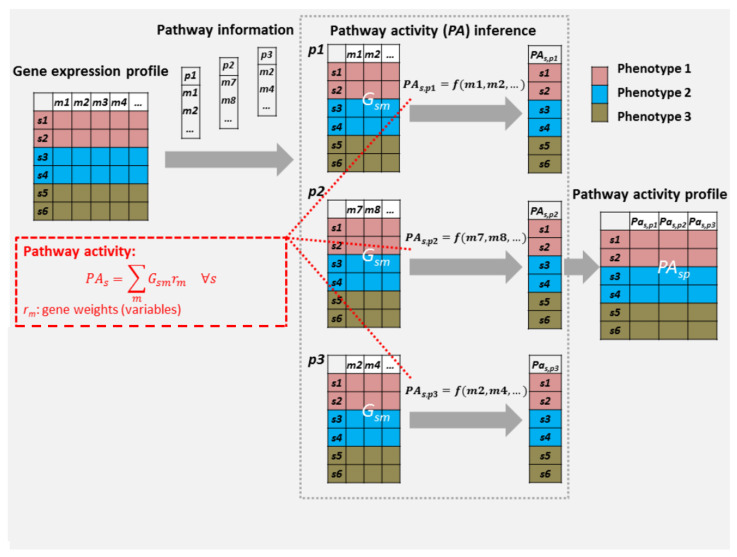
Pathway activity inference flowchart for DIOPTRA. The normalised RNA-Seq data and a number of pathway gene sets serve as the input to the pathway activity inference pipeline. For each pathway *p*, pathway activity values $pa_{sp}$ are calculated as linearly combined expressions of pathway genes $G_{sm}$ multiplied with gene weights $r_{m}$, where $r_{m}$ is decided by two variables ($rp_{m}$ and $rn_{m}$) in the model. A pathway activity profile is formed by connecting the pathway activity vectors of all pathways, and the new matrix of pathway activity profiles is obtained and further evaluated.

In this work, we propose an optimisation-based pathway activity inference model, DIOPTRA (Disease OPTimisation for biomaRker Analysis), which infers pathway activity in a supervised manner, i.e., by utilising clinical phenotype data and maximising the discriminative power of pathway activity values towards the sample subtype class. In DIOPTRA, pathway activity is created by linearly combining expressions of pathway constituent genes and then multiplying them with gene weights determined by the model. The objective function of the optimisation problem is used to minimise the distances between samples and their corresponding class intervals. Overall, DIOPTRA derives a pathway activity feature that allows samples with the same label to be clustered both together as well as further away from samples of different class; the overall procedure is summarised in Figure 1.

The pathway activity value $pa_{s}$ in the model is defined as a weighted linear summation of the expressions of pathway constituent genes:

(1) $$pa_{s}=\sum_{m}G_{sm}\cdot \left(rp_{m}-rn_{m}\right)\;\forall s$$

where $G_{sm}$ is the gene expression value for sample *s* and gene *m* for the current pathway *p*; furthermore, $rp_{m}$ and $rn_{m}$ are positive continuous variables that model the positive and negative weights of a gene *m*, and their values are determined by the optimisation model.

The following three equations set the restrictions on gene weights. Equations (2) and (3) ensure that for each gene *m* at most one of $rp_{m}$ and $rn_{m}$ can take positive values. This is implemented by introducing a set of binary variables $L_{m}$, which takes a value of 0 or 1:

(2) $$rp_{m}\leq L_{m}\;\forall m$$

(3) $$rn_{m}\leq \left(1-L_{m}\right)\;\forall m$$

when $L_{m}=1$, $rp_{m}$ can take any value between 0 and 1, while $rn_{m}$ is forced to be 0; otherwise, when $L_{m}=0$, $rp_{m}$ is forced to be 0, and $rn_{m}$ can be between 0 and 1. Overall, a gene can have positive, negative, or zero weight towards the composite feature ($pa_{s}$) construction. For normalisation purposes, the summation of absolute gene weights should be equal to 1:

(4) $$\sum_{m}\left(rp_{m}+rn_{m}\right)=1$$

On the pathway activity dimension, each sample phenotype occupies a unique interval and does not overlap with the intervals of other phenotypes, as implemented by Equations (5)–(7):

(5) $$UP_{k}+\varepsilon \leq LO_{c}+U\cdot \left(1-Y_{kc}\right)\;\forall k<c$$

(6) $$UP_{c}+\varepsilon \leq LO_{k}+U\cdot Y_{kc}\;\forall k<c$$

(7) $$UP_{c}-LO_{c}\geq \varepsilon \;\forall c$$

where both *c* and *k* denote phenotypes; $LO_{c}$ and $UP_{c}$ are continuous variables modelling the lower and upper bound of the activity interval of phenotype *c*, respectively; *U* and ε are arbitrarily large and small positive constants, respectively, where *U* ensures the non-overlapping nature of the class intervals and ϵ indicates the minimum distance between two classes; and $Y_{kc}$ is a set of binary variables used to ensure the non-overlapping of the pair-wise phenotype intervals. When $Y_{kc}=1$, the lower bound of phenotype *c* is greater than the upper bound of phenotype *k* ($LO_{c}>UP_{k}$), while when $Y_{kc}=0$, the lower bound of phenotype *k* is greater than the upper bound of phenotype *c* ($LO_{k}>UP_{c}$). In either case, the phenotype ranges of *k* and *c* do not overlap.

For sample *s*, the violation distance $D_{s}$ (positive continuous variable) is defined as the distance between sample pathway activity $pa_{s}$ and the pathway activity range of the phenotype that the sample belongs to $\left[LO_{c_{s}},UP_{c_{s}}\right]$.

(8) $$D_{s}\geq pa_{s}-UP_{c}\;\forall s,c_{s}$$

(9) $$D_{s}\geq LO_{c}-pa_{s}\;\forall s,c_{s}$$

**Figure 2:**
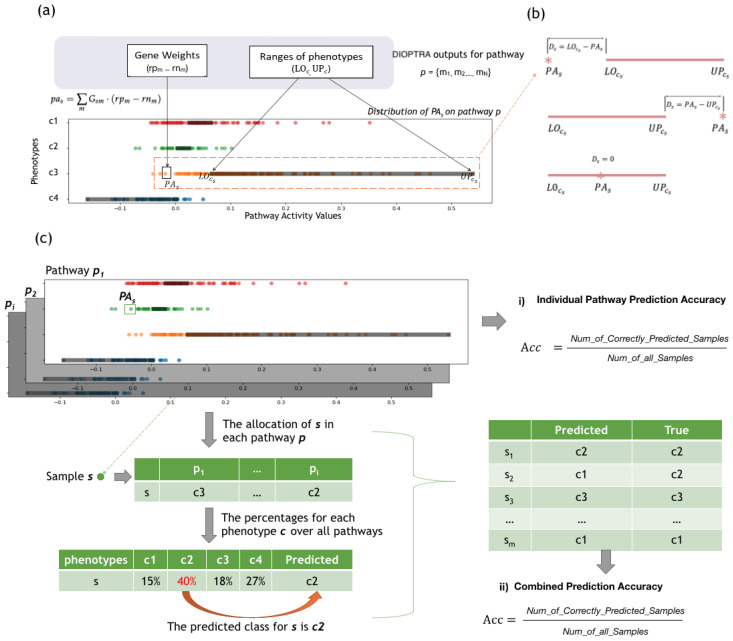
Individual Pathway Evaluation. (**a**) Motivating Example of the DIOPTRA model. DIOPTRA seeks to infer pathway activity as a discriminative feature for sample classes. Pathway activity ($pa_{s}$) is defined as the weighted linear summation of expressions of constituent genes *m*, where the gene weights $\left(rp_{m}-rn_{m}\right)$ are modelled as free variables. On the pathway activity dimension, each class occupies a distinct range ($\left[LO_{c_{s}},UP_{c_{s}}\right]$, represented by the black boxes), and classes do not overlap. The points indicate samples coloured according to class, while values are $pa_{s}$. (**b**) Three possible scenarios between $pa_{s}$ and $\left[LO_{c_{s}},UP_{c_{s}}\right]$. The distance between the $pa_{s}$ and its corresponding $\left[LO_{c_{s}},UP_{c_{s}}\right]$ is the sample violation distance $D_{s}$. (**c**) Two applications of the outputs of the DIOPTRA model. (i) Individual Pathway Prediction Accuracy: after $pa_{s}$ values are obtained, samples are allocated into the nearest class range, then accuracy for *p* is calculated as the percentage of samples that are allocated to the correct class. (ii) Combined Prediction Results: after obtaining sample allocations from all pathways, the predicted class for *s* is the one with the highest number of pathways; the combined accuracy is then calculated after dividing the number of correctly predicted samples by the total.

Figure 2b shows three possible scenarios between $pa_{s}$ and $\left[LO_{c_{s}},UP_{c_{s}}\right]$. When $pa_{s}$ is smaller than its lower bound $LO_{c_{s}}$, the violation distance becomes $D_{s}=LO_{c_{s}}-pa_{s}$, while when $pa_{s}$ is greater than its upper bound $UP_{c_{s}}$, the violation distance becomes $D_{s}=pa_{s}-UP_{c_{s}}$, and when $pa_{s}$ is inside its target range, the violation distance is equal to 0.

The objective function (10) in the model minimises the sum of violation distances $D_{s}$ over all samples:

(10) $$Min\;z=\sum_{s}D_{s}$$

This novel optimisation model, named DIOPTRA, consists of a linear objective function and several linear constraints. The presence of both continuous and integer variables yields a mixed-integer linear programming (MILP) model; the mathematical formulation is summarised below.

**Objective function:**

Minimising the summation of violation distance (10)

**Subjected to:**

Pathway activity definition (1)

Restrictions on positive and negative gene weights (2, 3 and 4)

Non-overlapping constraints for phenotype ranges (5, 6, and 7)

Sample violation distance calculation (8 and 9)

**Variables:**

$rp_{m},rn_{m},D_{s}\geq 0$;

$pa_{s},LO_{c},UP_{c}:Unstricted$;

$L_{m},Y_{kc}\in \left\{0,1\right\}$

Because MILP models are NP-hard, computational requirements may increase significantly as the number of binary variables increases [41]. DIOPTRA builds upon concepts introduced in our previous work (DIGS [23]) by eliminating most of the binary variables used previously. The DIGS model uses a set of binary variables to force the class intervals enclosing the samples. DIOPTRA removes this by replacing the sample enclose constraints with violation distance constraints. In addition, DIOPTRA releases limitations on the number of genes that can obtain gene weights, thereby eliminating another group of binary variables.

Figure 2a presents a motivating example to illustrate how DIOPTRA separates samples of different classes. The input data consist of a simulated pathway expression matrix, where 30 member genes from a specific pathway serve as columns and COAD samples serve as rows. The output consists of gene weights ($rp_{m}-rn_{m}$), which are further used for calculating the PA values via Equation (1), as well as the class intervals $LO_{c}$ and $UP_{c}$. The points in Figure 2a represent the inferred pathway activity values of all samples, while the black boxes on the horizontal axes represent the class intervals (here, the four cancer subtypes of COAD). All the intervals on the plot are non-overlapping, as they are restricted by Equations (5)–(7). With the violation distance $D_{s}$ calculated by Equations (8) and (9), the objective function (10) performs the task of minimising the summation of $D_{s}$ for all samples. As shown in Figure 2a, the pathway activity values for samples of the same class label are clustered together. The bounds of each class are close to the centres of the sample clusters, which illustrates the ability of the model to separate samples of different phenotypes to the largest extent.

The MILP model is solved using the general purpose CPLEX solution algorithm implemented in the General Algebraic Modelling System (GAMS) [40]. Solutions are identified in a user-specified time limit (200 seconds by default). Note that simply increasing the time limit only marginally improves the quality of the solution. Therefore, both the feasible solution and the optimal solution are acceptable. In addition, the relative optimality criterion solver (optcr) is set to its default value of 0, that is, the limit for Gap, which reflects the percentage difference between the best potential target and the best found target, is not restricted in the model solver report.

### 2.3. Comparison of Pathway Activity Inference Approaches

To demonstrate the efficiency of the proposed DIOPTRA model for pathway activity inference, we implemented a range of other pathway activity inference methods for comparison, including two baseline methods: (i) the approach of Guo et al. [14] (referred to as MEAN), which calculates average gene expression values as pathway activity and is the original work that proposed the concept of applying dimensionality reduction based on functional gene sets; (ii) the approach of Bild et al. [26] (referred to as PCA), which calculates the top principle component as a representation of the pathway activity. This method has been adopted in many studies [16,17,18,19].

In addition, three more advanced methods were used for benchmarking DIOPTRA: (i) our previous model, DIGS [23], which selects the best subset of genes to build pathway activity while minimising the number of misclassified samples; (ii) the GSVA [42] approach, a variation of Gene Set Enrichment Analysis [43]; and (iii) the Pathifier [19] approach, which investigates the extent to which the behavior of a pathway deviates from normal. Pathifier was shown to be the most competitive method in a recent review of pathway activity inference methods [44].

### 2.4. Method Evaluation

Three metrics were used to evaluate comparisons of pathway activity inference methods: (i) sample prediction accuracy using machine learning classifiers, (ii) robustness against noise in gene expression data, and (iii) ability to use pathway activity values for survival analysis. The comparison carried out via these metrics is described below.

#### 2.4.1. Classification

The accuracy of classifying samples to the appropriate class using the pathway activity matrix was used to assess the ability of pathway activity vectors to separate samples of different phenotypes. In this work, we used Random Forest (referred to as RF) and K-Nearest Neighbor (referred to as KNN). To obtain robust results and assess the generalisation properties of the trained models, ten-fold stratified cross-validation was used. To further assess the reproducibility of the results, we ran each cross-validation analysis three times, generating 30 training and 30 testing sets. The two classifiers were trained on the pathway activity profile inferred from the training samples and tested on the pathway activity profile inferred from the testing samples.

Binary classification (sometimes referred to as two-class classification) and multi-class classification were both implemented. Binary classification uses tumour and normal sample labels, while multi-class classification refers to the classification of molecular subtypes (Table 1). To avoid the effects of unbalanced sample groups for two-class labels in both datasets, SMOTE [45] was applied to the training sets to ensure that the number of normal samples was balanced to the number of tumour samples. The RF parameter *n_estimators* was tuned from 200 to 2000 using grid search optimisation on the training sets to optimise the performance of the trained model. As opposed to RF, KNN represents a basic and naïve classifier. The *k* parameter for KNN was selected using a trial-and-error process on the training dataset, testing values of 3, 5, 10, 20, 30, and 50. We selected *k* = 30, as it was the value providing the highest performance on the classification metrics. Both RF and KNN were implemented using the Python library *sklearn 0.24.0* [46]. The performance of the classifiers was evaluated using four metrics, namely, Accuracy, F1-score, Precision, and Recall, which were averaged over the 30 testing sets.

#### 2.4.2. Robustness against Noise in Data

The robustness of pathway activity inference methods when facing unpredictable fluctuations in gene expression values was assessed, with the expectation that pathway activity inference methods should be able to retain good classification performance as the noise level increases. The robustness evaluation procedure consisted of three steps: simulating noise in expression data, pathway activity inference on the simulated data, and evaluating classification accuracy. The pipeline is illustrated in Supplementary Figure S1. The gene expression perturbation simulation process was performed by permuting the sample order for the randomly selected genes [47]. The proportions of affected genes were set to 0%, 3%, 10%, and 50%, with increasing noise when the percentage increased. Therefore, in addition to the original gene expression profile (0%), three perturbed expression profiles were created for the two datasets. Finally, the robustness of the methods was evaluated using the multi-class classification accuracy as assessed by the KNN classifier.

#### 2.4.3. Survival Analysis

Survival analysis is a key means of expressing prognostic value in cancer studies. As a new composite feature that can aggregate the gene expression values, pathway activity values are expected to perform better at predicting survival than random data. As a new composite feature that aggregates the gene expression values, pathway activity values are expected to perform better than random data in predicting survival probabilities. In this work, the pathway activity data produced for cancer molecular subtypes (multi-class) was used to train the survival model with the sample clinical information.

Clinical data of both datasets were downloaded together with the raw counts data from the TCGA database. The event (“0” for observed death or “1” for not observed death) and duration time for samples were extracted from the clinical data. Considering the inaccuracy caused by the loss of clinical information, samples with too short a follow-up time (where the duration of the time was less than one year and the event was “0”) were removed. After sample selection, 196 samples (98 samples with label ’1’) were kept for COAD and 206 samples (103 samples with label “1”) were kept for BRCA.

The Survival Random Forest [48] model (implemented using the Python package *scikit-survival 0.16.0* [49]) was used for training and testing the survival model, and the concordance index (c-index) [50] was used for evaluating the model. On random data, the c-index was 0.5, meaning that values higher that 0.5 indicate good survival prediction.

### 2.5. Sample Classification through DIOPTRA

#### 2.5.1. Pathway and Gene Ranking

One of the most important applications of pathway activity inference is identifying significant pathway signatures related to cancer subtypes. Previous research has used computational or statistical approaches, such as p-value ranking or information gain index [30,42,51,52], to select the pathways with relatively high significance. In this work, benefiting from the explainability of the optimisation model, we present a comprehensive method that can identify pathways important for disease classification directly from the outputs of DIOPTRA, rather than relying on post-processing of pathway activity values through machine learning classifiers.

Figure 2a,b illustrates the mechanism of how DIOPTRA classifies the samples in individual pathways. Based on this, another application of the outputs of the DIOPTRA model is introduced in Figure 2c. For each pathway, the DIOPTRA model provides the pathway activity value for each sample *s* and the class ranges for each class *c*. By calculating the distance of samples to each class (violation distance $D_{s}$), samples can be allocated to their nearest class. In other words, the allocation result is the predicted class of the sample *s*, and the prediction accuracy of the pathway *p* can be calculated as the number of true positive samples divided by the total amount of samples, termed as the *Individual Pathway Prediction Accuracy*. Consequently, pathways can be ranked according to their ability to separate sample subtypes, and pathways with higher prediction accuracy are the ones most significant for disease classification.

In addition to ranking pathways, DIOPTRA provides quantitative evaluation and ranking of member genes of pathways. As the pathway activity value is defined by the weighted summation of gene expression values, a gene that obtains a higher weight ($rp_{m}-rn_{m}$) from the DIOPTRA model has a higher influence on pathway activity values. Moreover, as $rp_{m}-rn_{m}$ can be either positive or negative, the impact of the gene is decided by the absolute gene weights. Therefore, the final ranking of genes is decided by accumulating the absolute weights from all the available results (30 times model training) for the same pathway.

These DIOPTRA-identified important pathways were verified by SHapley Additive exPlanations (SHAP) analysis [53], which is a unified framework used to interpret the predictions of machine learning classifiers by assigning importance values to input features. We applied the *Tree Explainer* from the Python package *SHAP* [54] to our trained RF classifiers. The results of this SHAP analysis provide the pathways with the highest impact on the prediction accuracy of the RF.

#### 2.5.2. Assessing Sample Classification through DIOPTRA

Typically, pathway activity inference methods are assessed based on how well samples can be classified using machine learning classifiers on pathway activity values. However, as the DIOPTRA model can be used to predict the sample class directly, we illustrate its classification power here without resorting to the use of external classifiers.

Sample allocation in individual pathways can be combined to produce a final sample prediction; this process is illustrated in the two tables on the left-hand-side of Figure 2c. The first table shows the allocated class of sample *s* of each pathway *p*, while the second table counts the percentages for each class *c*. The percentage value represents the number of pathways that allocate sample *s* into class *c*, and the combined prediction class for sample *s* is the class with the highest percentage. As in the example shown in Figure 2, the highest percentage is 40%, which means that in 40% of KEGG pathways sample *s* is allocated to class c2. Therefore, the combined prediction class for *s* is c2. The table on the right-hand-side of Figure 2c shows the accuracy calculation of the combined prediction for all samples, which is determined as the number of correctly predicted samples divided by the number of all samples.

## 3. Results

### 3.1. Classification Comparison

**Figure 3:**
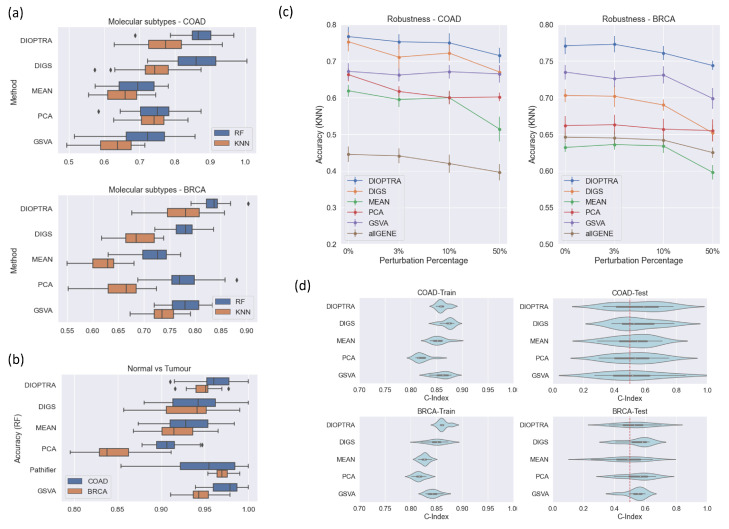
Assessment of pathway activity inference methods. (**a**) Comparison of pathway activity inference prediction performance by Random Forest (RF) and K-Nearest Neighbour (KNN) on the multi-class scenario. (**b**) Prediction performance comparison on the two-class scenario, including the Pathifier method, which is designed for binary labelled data. (**c**) Prediction accuracy (multi-class scenario) with noise introduced to the RNA-Seq data. The x-axis represents the percentage of samples perturbed and the y-axis indicates the prediction accuracy and standard errors of KNN over one instance of ten-fold cross-validation. AllGENE refers to directly using the gene expression values to train and test the classifier. (**d**) C-index for evaluating the Random Survival Forest model fitted on the pathway activity (multi-class scenario); C-index = 0.5 indicates the performance of a random classifier.

Classification performance comparisons were conducted for both two-class and multi-class problems. The pathway activity profiles inferred by six methods (DIOPTRA, DIGS, MEAN, PCA, GSVA and Pathifier) were input to the RF and KNN classifiers for training. Note that Pathifier was used only for two-class classification problems, as it is not designed for multi-class problems. It should be noted that in order to achieve an objective evaluation of classification performance, the training process (i.e., inferring pathway activity and classifier training) was always blind to testing. Classification evaluation metrics were averaged across the testing sets and classification accuracies of all methods; the classifier combinations are shown in Figure 3a,b for multi-class classification and two-class classification, respectively. The outcomes of all four classification metrics are provided in Supplementary Table S1.

From Figure 3a, it is obvious that the inferred pathway activity using the proposed DIOPTRA model results in more accurate overall predictions than the methods in the literature. In both datasets and classifiers, DIOPTRA manages to achieve higher average accuracy towards the classification of molecular subtypes (multi-class problem). For the two-class classification problem (Figure 3b), although GSVA performs best for BRCA and Pathifier performs best for COAD, DIOPTRA is the second-best method in both datasets. However, as all methods perform well in two-class classification problems (over 90% accuracy, except for MEAN on BRCA), the slight differences between methods are negligible. Therefore, overall, DIOPTRA shows strong superiority on cancer classification, especially in multi-class scenarios. Furthermore, as can be seen in Supplementary Table S1, the DIOPTRA model has better prediction results than the other methods in terms of F1-score, Precision, and Recall in the majority of cases.

### 3.2. Robustness Comparison

Robustness was evaluated by calculating the multi-class classification accuracy on RNA-Seq datasets with the data perturbed at various levels. The KNN classifier was used (where *k* = 30), and the accuracy was calculated by averaging across testing sets. The results are shown in Figure 3c. In addition to the pathway activity inference methods, a gene-level approach, referred to as allGENE, is used here, with gene expression values used to train and test with the KNN classifier. This serves as a baseline for evaluating the extent to which simulated noisy data can affect sample classification accuracy.

As seen in Figure 3c, DIOPTRA provides the best overall performance. As more noise is introduced to the data, accuracy in DIOPTRA remains above 70% for both COAD and BRCA. Although PCA is able to maintain more stable performance when the perturbation degree is increased, its comparatively low accuracy in non-perturbed datasets inversely demonstrates its poor power in differentiating sample types. For the performance of GSVA, the accuracy in COAD does not fluctuate as perturbation increases; however, it shows a decreasing tendency in BRCA. This phenomenon can be explained by the nature of the GSVA method. GSVA focuses on the ranking of the genes rather than their expression values. Therefore, the simulated perturbation process (i.e., re-ordering part of the expression values) can take the form of either slight effects or obvious effects on the gene rankings.

### 3.3. Survival Comparison

Survival regression was conducted for both the training and testing sets. The C-index results are reported in Figure 3d. For the training sets, all methods perform well on the two datasets. For the COAD dataset, DIGS takes the first place, while for BRCA DIOPTRA takes the first place. In the testing sets, nearly all methods gain a C-index over 0.5, except for the MEAN method on BRCA. Although the differences between the five pathway activity inference methods are not particularly distinct, DIOPTRA and DIGS show greater performance in predicting survival information than the other methods.

**Figure 4:**
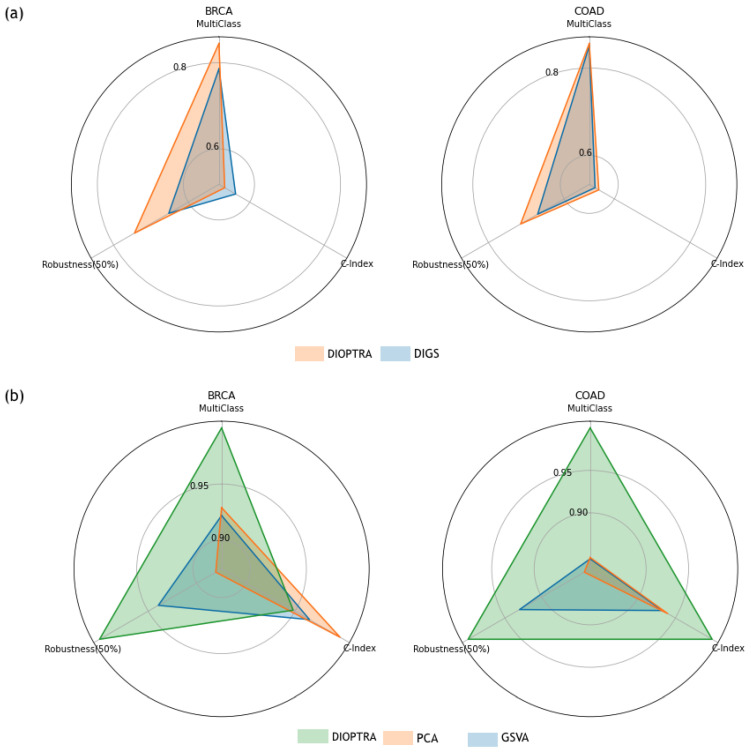
Radar charts; each vertex counter-clockwise from 12 o’clock of the radar chart indicates sample phenotype prediction accuracy on molecular subtypes, robustness against 50% perturbed genes, and survival regression as evaluated by C-index, respectively. (**a**) Comparison of the performance between DIOPTRA and the original DIGS. (**b**) Comparison of the performance between three representative methods (DIOPTRA, PCA, and GSVA). The number on each vertex is scaled to 0-1 for these two plots.

Figure 4 provides a comprehensive overview of comparisons. The multi-class prediction vertex uses the RF classification accuracy for the multi-class scenario. The robustness (50%) vertex is the KNN classification accuracy produced on a 50% perturbed dataset, while the c-index vertex represents the c-index values produced using testing samples. Two-class classification accuracy is not included, as the results for all methods were similar. The values on each vertex were normalised using a 0–1 scale to make the differences between methods clearer. Figure 4a compares the performance between DIOPTRA and DIGS. The shadowed area of DIOPTRA is larger than that of DIGS, indicating that the DIOPTRA model has better quality pathway activity than the previous optimisation formulation in DIGS. Figure 4b compares three pathway activity inference methods that are representative of the different calculation principles, i.e., DIOPTRA for optimisation, PCA for dimensionality reduction, and GSVA for gene ranking. DIOPTRA outperforms the other methods for nearly all the metrics on both datasets, especially in the case of multi-class prediction.

### 3.4. DIOPTRA Prediction Performance and Identification of Biologically Relevant Pathways

The prediction accuracy of DIOPTRA by itself (see Section 2.5.2) is comparable to that achieved when DIOPTRA-derived pathway activity vectors are used for classification with standard machine learning classifiers. Table 2 compares the performance of DIOPTRA itself with DIOPTRA + Machine Learning Classifiers. All accuracy values are produced for multi-class classification scenarios. It is shown that the accuracy of DIOPTRA is not significantly degraded compared to DIOPTRA + KNN, which means that DIOPTRA by itself is competitive with simple machine learning classifiers. Therefore, the powerful classification ability of the DIOPTRA model is confirmed.

From another point of view, the prediction accuracy of DIOPTRA by itself can be compared to accuracies produced by the other pathway activity inference methods using machine learning classifiers. The accuracy of DIOPTRA in Table 2 is at the same level as the other methods + KNN in Figure 3a. In detail, the DIOPTRA accuracy for BRCA subtypes is 0.67, while the accuracy of other moethods is between 0.6 and 0.7; moreover, the accuracy of DIOPTRA for COAD subtypes is 0.75, while for other methods it is between 0.65 and 0.75. This implies that DIOPTRA by itself can achieve the classification performance of other widely used pathway activity inference methods.

We ranked KEGG pathways according to their Individual Pathway Prediction Accuracy calculated using DIOPTRA. The member genes of each pathway were ranked according to their gene weights, and final rankings were aggregated using the results of ten-fold cross-validation. Table 3 shows the top ten pathways for BRCA along with the top five pathway constituent genes and their accumulated weights. The ranking of all pathways and genes for BRCA and COAD are listed in Supplementary Table S2.

**Figure 5:**
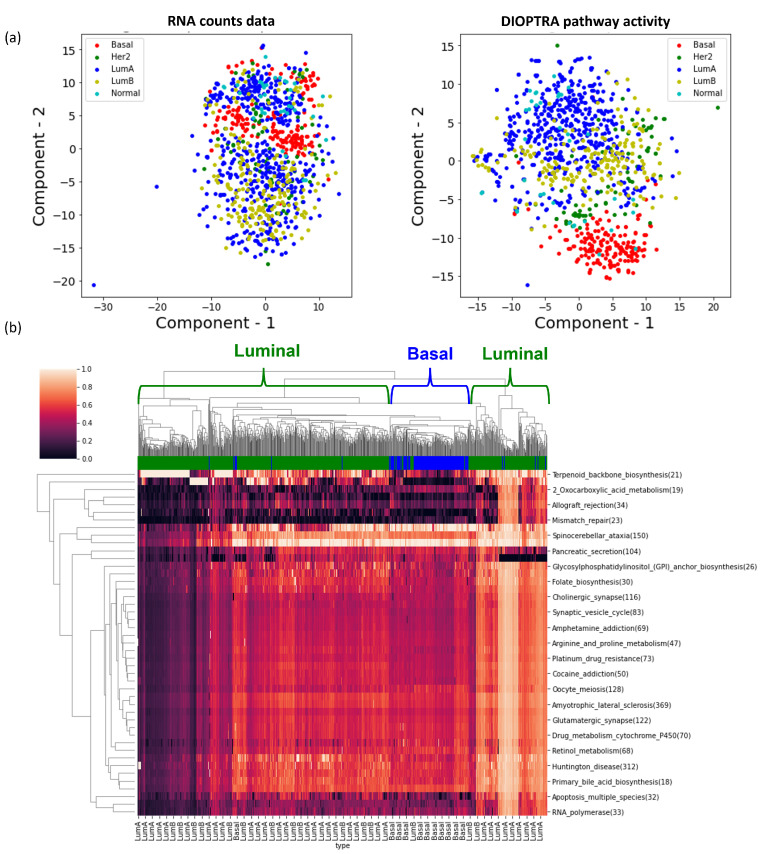
Visualisations of significant pathways in BRCA. (**a**) The left tSNE plot is generated from the RNA count data with genes across all KEGG pathways, while the right scatter plot is generated from the activities for KEGG pathways derived from the DIOPTRA model using all samples. (**b**) The hierarchical clustering heatmap represents pathway activities of the top 70 pathways with the highest prediction accuracy in DIOPTRA.

To demonstrate the effectiveness of the highly-ranked pathways, we projected the pathway activity values of the top quartile pathways (i.e., 70 pathways) in two-dimensional visualisation using tSNE. In Figure 5, the tSNE plot that clusters the samples using pathway activity shows greater refinement of cancer subtypes compared to the projection of RNA-Seq data. Especially for subtypes with a large number of samples, such as Luminal A, Luminal B, and Basal (see Table 1), the clusters are much clearer. The hierarchical clustering heatmap using the pathway activity values verifies this observation as well. We repeated the same analysis for the remaining pathway activity inference methods and COAD dataset, with the results presented in Supplementary Figure S2. Overall, the performance of DIOPTRA is the best and DIGS takes second place. The other methods do not show improvements compared to the RNA-Seq data.

## 4. Discussion

### 4.1. Computational Efficiency Improvements in DIOPTRA

To demonstrate the improvements in computation efficiency with DIOPTRA, we summarised the Model Status and the Gap value from all optimisation model solutions. Both DIOPTRA and DIGS models were solved 30 times for each pathway (three ten-fold cross-validations), resulting in a total amount of solutions equal to 8370 for each dataset and each model.

For both datasets, a total of 16,740 MILPs (8370 per dataset) were solved. It was found that DIOPTRA yields more Optimal and Integer (feasible) solutions than DIGS (660 more Optimal solutions for COAD and 90 more Integer solutions for BRCA), indicating that DIOPTRA can achieve a better quality of solutions. The Gap value metric, which indicates the closeness of the solution achieved to the best possible value, is plotted in histograms (Supplementary Figure S3), showing that DIOPTRA provides a considerable improvement in terms of decreasing gap values. For the smaller datasets (COAD), the distribution of Gap values of DIOPTRA is in the range of [0, 1], and nearly half of the Gap values are less than 1, while for DIGS nearly all the Gap values are 1. For the more computationally intensive dataset (BRCA), although most Gap values with both methods are equal to 1, the number of Gap values less than 1 is larger for DIOPTRA than DIGS.

### 4.2. Exploration of Top-Ranked Pathways

To validate the pathways that were identified as important Table 3, we looked to the literature to find the connections between these pathways, genes, and breast cancer. Several studies have demonstrated the role of Circadian Rhythm as an effective tumour suppressor [55,56,57]. The development of tumours is triggered by the stimulation or disruption of signalling pathways at the cellular level as a result of the interaction between cells and environmental stimuli. The Circadian Rhythm pathway synchronises the timekeeping in the peripheral tissues by integrating the light–dark input from the environment. The ROR family of genes, which is highly ranked by DIOPTRA, affect the Circadian Rhythm pathway regulation through regulating the secondary transcription/translational feedback loop [57]. Peroxisome protein levels or enzymatic activities of peroxisome metabolism are largely reduced in breast tumours [58]. The top constituent gene, AGXT, is highly involved in colorectal cancer [59] and hepatocellular carcinoma [60]. In addition, the relationship between folate and the risk of breast cancer has been greatly investigated [61,62]. It is noticeable that the GSTM family genes play a crucial role in chemical carcinogenesis, platinum drug resistance, drug metabolism cytochrome P450, and drug metabolism of other enzymes pathways. We found that the importance of the GSTM family to the risk of breast cancer has been verified by several studies [63,64,65,66].

The two highly ranked pathways in Table 3, namely, pancreatic secretion and chemical carcinogenesis, are not found to have a direct association with breast cancer in the literature. Moreover, their top-ranked genes are not found to be directly related to cancers. However, the ranking of the pathways is the result of the synergistic action of all the member genes. Therefore, using protein–protein interaction (PPI) networks to find the genes that are physically close to the top-ranked genes is another way to understand the pathways. To understand the functions of these two pathways, we performed further analysis by reconstructing and clustering their corresponding PPI networks. In this study, the PPI data were collected from the STRING database [67], and the MCL algorithm [68] was used for clustering. The clusters were visualised in Supplementary Figure S4 using Cytoscape [69], where the size of the nodes is proportional to their weight as identified by DIOPTRA and the edges are proportional to the interaction confidence score. In principle, proteins in the same cluster are either part of the same protein complex or perform a similar molecular function.

For the pancreatic secretion pathway, the gene with the highest weight, CA2, is the most active enzyme found in nature [70]. The cluster shows that CA2 interacts with a large number of SLC family member genes in the PPI network. Therefore, it can be assumed that the role of SLC family genes is essential for high ranking in the pancreatic secretion pathway, while the SLC gene family is found to be highly correlated with breast cancer [71]. For the chemical carcinogenesis pathway, the previously described GSTM family genes are abundantly present in the GSTM5-centered cluster. The ADH and ALDH gene families that appear in this cluster are risk factors for many types of cancer, and have abnormal activity in stage IV breast cancer [72].

Furthermore, the top 30 pathways identified by SHAP are presented in Supplementary Figure S5; the top ten pathways for each molecular subtype are presented in Supplementary Figures S6 and S7. From the results, significant pathways identified by the DIOPTRA model overlap highly with pathways that have a significant impact on the RF classifier. Over half of the top 30 pathways from SHAP overlap with the top 70 pathways of the DIOPTRA pathway ranking list. In addition, SHAP identifies other highly relevant pathways for BRCA, such as the Wnt Signaling pathway, AMPK signaling pathway, and Breast Cancer pathway, which means that the other way of explanation for the pathway activity values inferred by DIOPTRA can provide more information on revealing the disease pathway markers.

## 5. Conclusions

Integrating reliable pathway information into analysis of high-throughput omics data has been implemented in various studies. Such approaches have become popular in disease classification tasks using bulk profiling data, and have attracted attention in the field of single-cell RNA-Seq analysis. Most of the existing pathway activity inference methods face limitations in either not being applicable for multi-classification scenarios or lacking biological interpretability. Our previous work introduced a mathematical model, DIGS [23], which to the best of our knowledge is the only optimisation-based model that infers pathway activity values in a supervised manner and is suitable for multi-class tasks. The strong performance of the DIGS model motivated us to expand it by further improving its accuracy and interpretability. Therefore, in this work we present an MILP mathematical programming model, called DIOPTRA, that has better optimisation solution qualities and is more computationally efficient.

DIOPTRA either outperforms or performs on par with other widely used pathway activity inference methods. We evaluated the pathway activity methods using multiple metrics in both binary and multi-class scenarios, and additionally performed survival and robustness analyses. DIOPTRA shows competitive performance on these evaluation metrics, achieving particularly outstanding results in multi-class scenarios. The strong performance of DIOPTRA on multi-class problems, including sample classification, model robustness, validation, and survival analysis, highlights its advantages over other existing methods, particularly for multi-class problems. A key property of modelling in DIOPTRA is its interpretable nature in deriving rules that enable pathway and gene prioritisation. The application of DIOPTRA to RNA sequencing data for breast and colon cancers permitted the identification of accurate diagnostic and prognostic models for these clusters based on their assessment using stratified cross-validation. Despite the fact that these models outperform other pathway inference based methods as well as the method using all genes as input to classification models, further exploration via application of these or similar models for diagnostic and prognostic purposes in independent retrospective and prospective datasets remains instrumental in validating their translational potential in clinical settings.

## Figures and Tables

**Table 1:** TCGA dataset details.

Dataset	Tumour or Normal Label	Molecular Subtype Label
COAD	Tumour: 480 Normal: 41	CMS1: 85
		CMS2: 165
		CMS3: 58
		CMS4: 120
BRCA	Tumour: 1091 Normal: 120	LumA: 579
		LumB: 217
		Basal: 191
		Her2: 82
		Normal-Like: 22

**Table 2:** Prediction accuracy achieved by DIOPTRA and DIOPTRA + Machine Learning Classifiers.

Classifier	BRCA	COAD
DIOPTRA	0.67 (0.064)	0.75 (0.043)
DIOPTRA+KNN	0.74 (0.046)	0.76 (0.068)
DIOPTRA+RF	0.84 (0.031)	0.85 (0.060)

**Table 3:** Significant pathways and genes for BRCA.

KEGG Pathway Name	No. Gene	Top Genes and Weights ^1^									
Pancreatic secretion	104	CA2	4.36	CPA1	0.72	CHRM3	0.69	PRSS2	0.53	CPA2	0.33
Circadian rhythm	32	RORB	3.24	ROR1	0.78	PRKAA2	0.49	RORA	0.49	CUL1	0.47
Peroxisome	87	AGXT	5.39	HAO2	3.21	ACSL6	0.23	PEX11A	0.10	IDH2	0.08
Chemical carcinogenesis	81	GSTM5	6.07	PTGS2	0.71	GSTA1	0.66	GSTA2	0.44	CYP1A1	0.30
Platinum drug resistance	73	GSTM5	6.71	GSTA2	1.30	GSTA1	0.82	CDKN2A	0.21	GSTT2B	0.15
Drug metabolism cytochrome P450	70	GSTM5	6.33	GSTA1	0.91	GSTA2	0.69	UGT2B11	0.22	FMO2	0.21
Folate biosynthesis	30	TPH1	4.11	PAH	3.88	ALPL	0.79	MOCOS	0.34	FPGS	0.20
Drug metabolism other enzymes	79	GSTM5	5.98	GSTA1	0.93	GSTA2	0.65	XDH	0.55	GSTT2B	0.21
Cocaine addiction	50	SLC18A2	3.56	DRD1	1.16	GRIN2A	0.93	CREB3L3	0.51	SLC18A1	0.41
Carbon metabolism	118	AGXT	3.468	HAO2	2.178	ALDOB	1.962	PHGDH	0.605	PSAT1	0.147

^1^ The gene weights are summations from 30 model trainings (three ten-fold cross-validations).

## Data Availability

The results shown here are based in whole or in part upon data generated by the TCGA Research Network: https://www.cancer.gov/tcga (accessed on 17 July 2021) and the KEGG: https://www.kegg.jp/kegg/ (accessed on 21 March 2022).

## Supplementary Materials

The following supporting information can be downloaded at: https://www.mdpi.com/article/10.3390/cancers15061787/s1, Figure S1: Robustness Evaluation Pipeline for Pathway Activity Inference Methods; Figure S2: tSNE dimension reduction scatters plots and Hierarchical clustering map of significant pathways in COAD; Figure S3: Computational efficiency Comparison between DIGS and DIOPTRA; Figure S4: Protein-protein interaction network for pancreatic secretion and chemical carcinogenesis; Figure S5: Top 30 pathway from SHAP for BRCA and COAD; Figure S6: Top 10 pathways from SHAP for each subtype of BRCA; Figure S7: Top 10 pathways from SHAP for each subtype of COAD; Table S1: Classification Metrics Comparison of RF and KNN for Pathway Activity Inference Methods; Table S2: The ranking of all pathways and genes for BRCA and COAD. All authors have read and agreed to the published version of the manuscript.
