# Supplementary material for: Optimisation Models for Pathway Activity Inference in Cancer
Source: Cancers (Basel). 2023 Mar 15;15(6):1787. doi: 10.3390/cancers15061787 (PMC10046797; doi:10.3390/cancers15061787)
Supplement: Supplementary file 1 [file cancers-15-01787-s001.zip › cancers-2176060-supplementary.pdf]

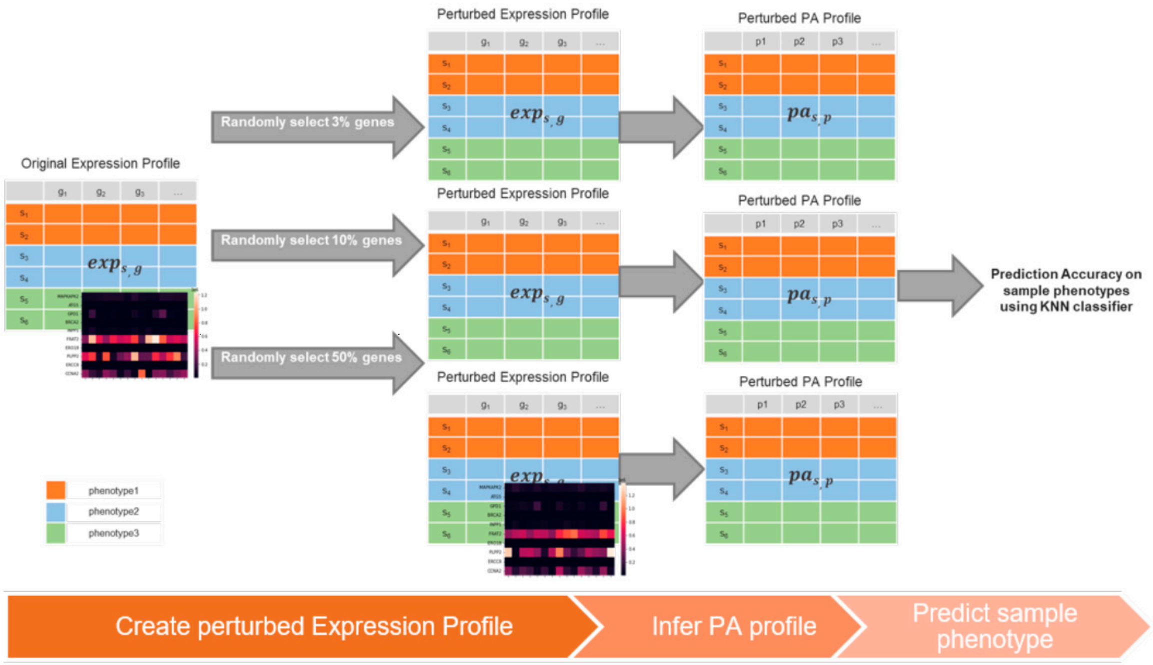

Figure S1. Robustness Evaluation Pipeline for Pathway Activity Inference Methods.

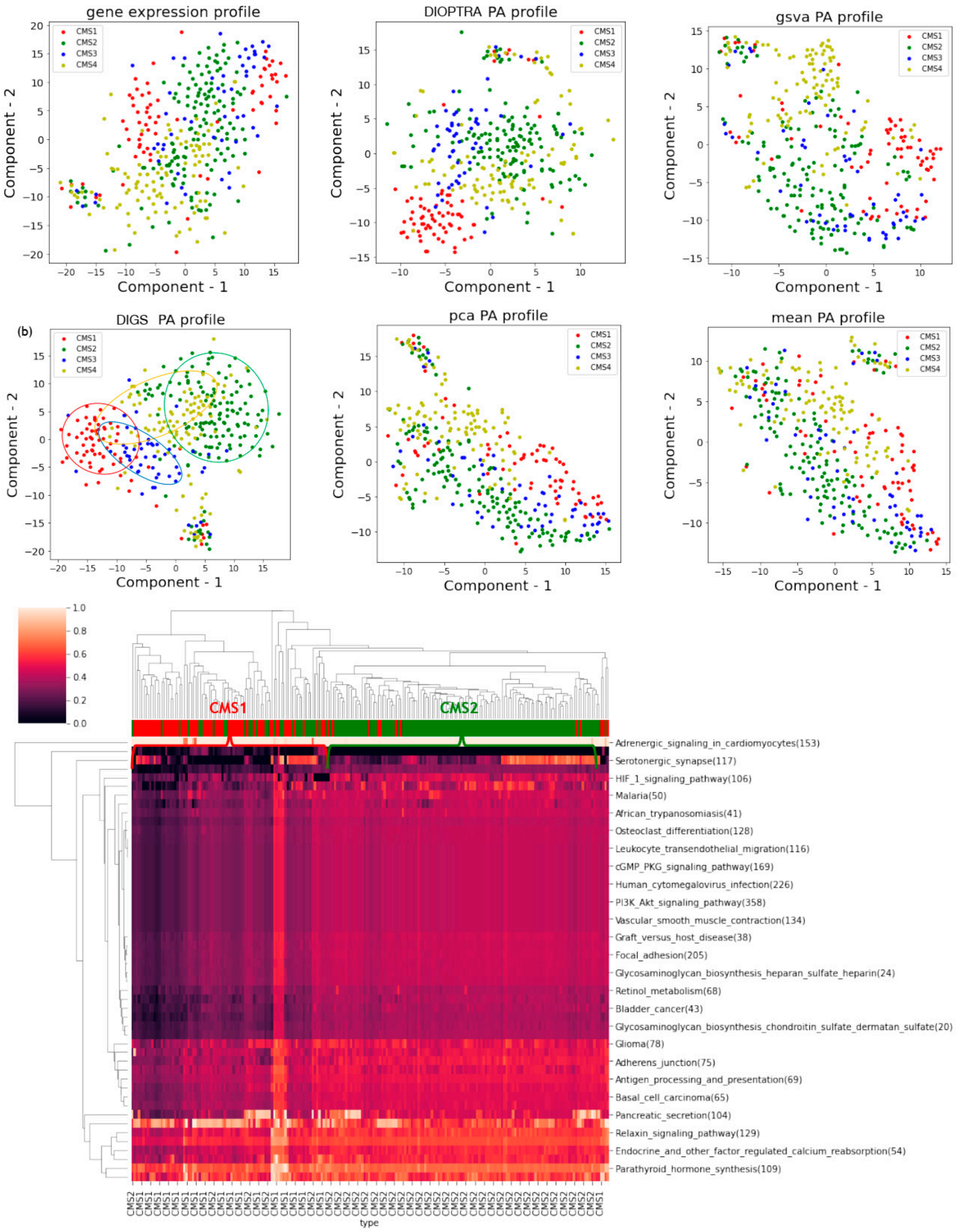

Figure S2. Pathway activity dimension reduction plots for PA inference methods and Hieratical clustering map of significant pathways (COAD).

COAD (480 samples, 4 subtypes)		
Solve status	DIGS	DIOPTRA
Optimal	0	660
Integer Solution	8370	7710
No Solution Returned	0	0

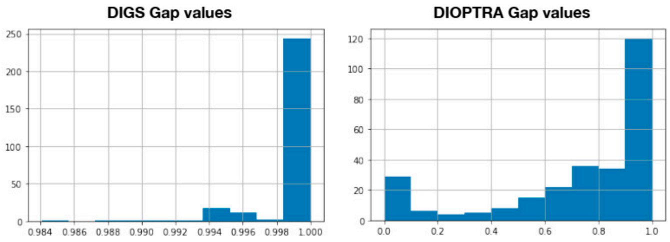

BRCA (1091 samples, 5 subtypes)		
Solve status	DIGS	DIOPTRA
Optimal	0	0
Integer Solution	8280	8370
No Solution Returned	90	0

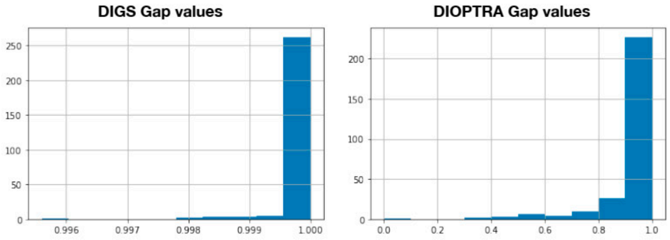

Figure S3. Calculation efficiency Comparison.

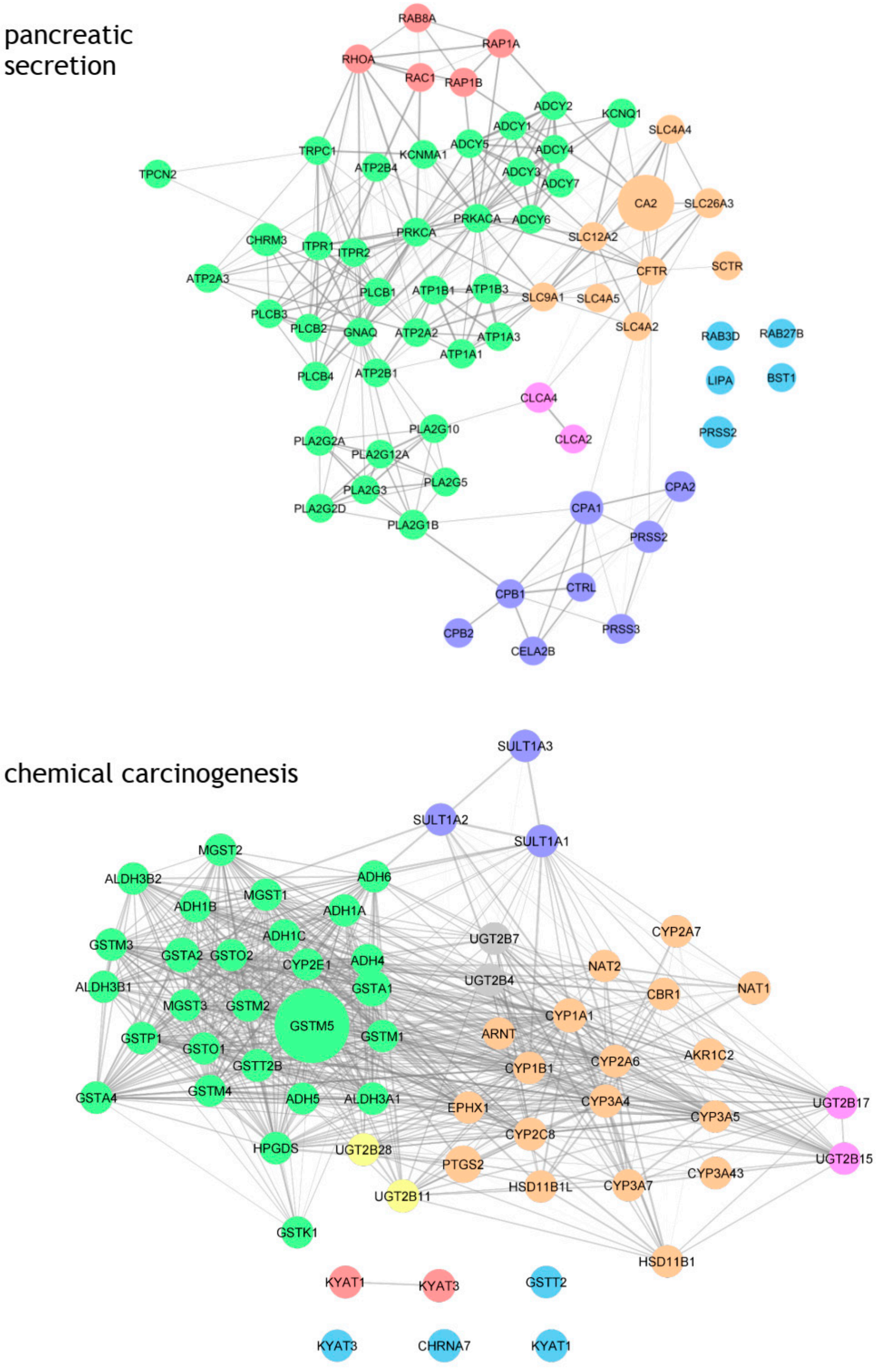

Figure S4. Protein-protein interaction network for pancreatic secretion and chemical carcinogenesis. The Protein-protein interaction networks are retrieved the protein-protein interaction network from the Stringdb for the proteins of each pathway. Proteins are clustered using MCL algorithm. The size of nodes are proportional to the weight identified by DIOPTRA and the edges are proportional to the interaction confidence score.

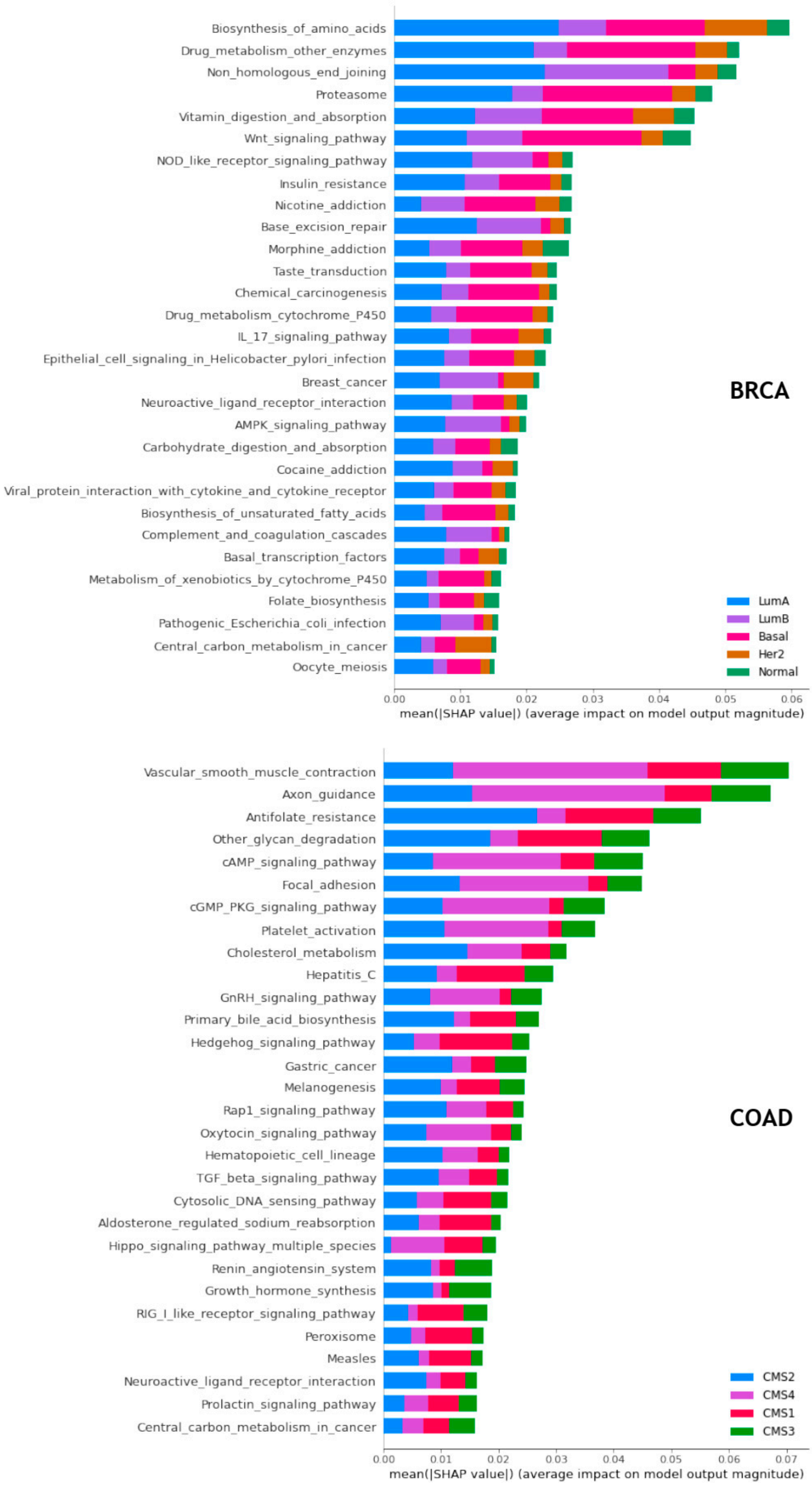

Figure S5. Top 30 pathway from SHAP for BRCA and COAD.

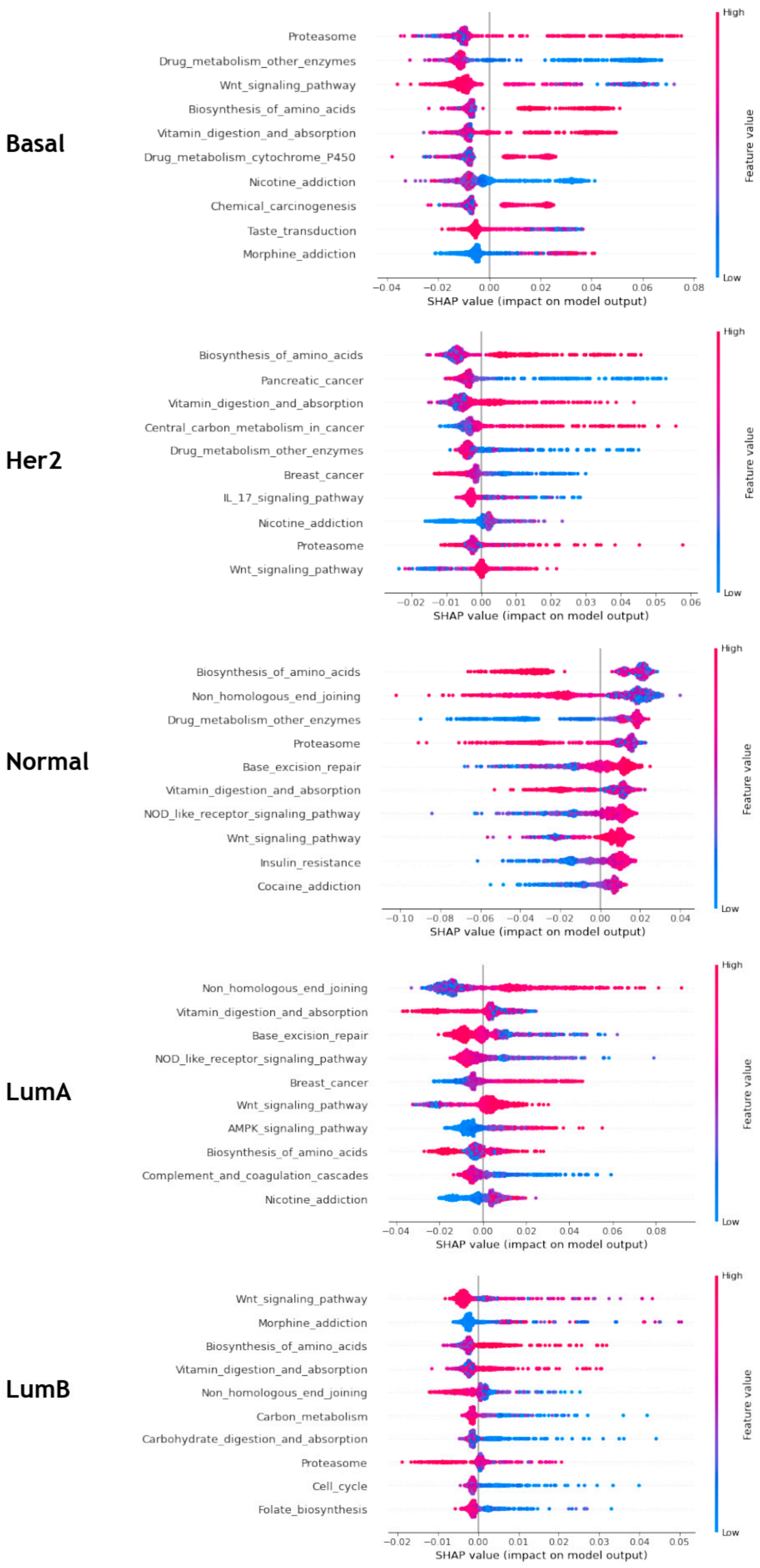

Figure S6. Top 10 pathways from SHAP for each subtype of BRCA.

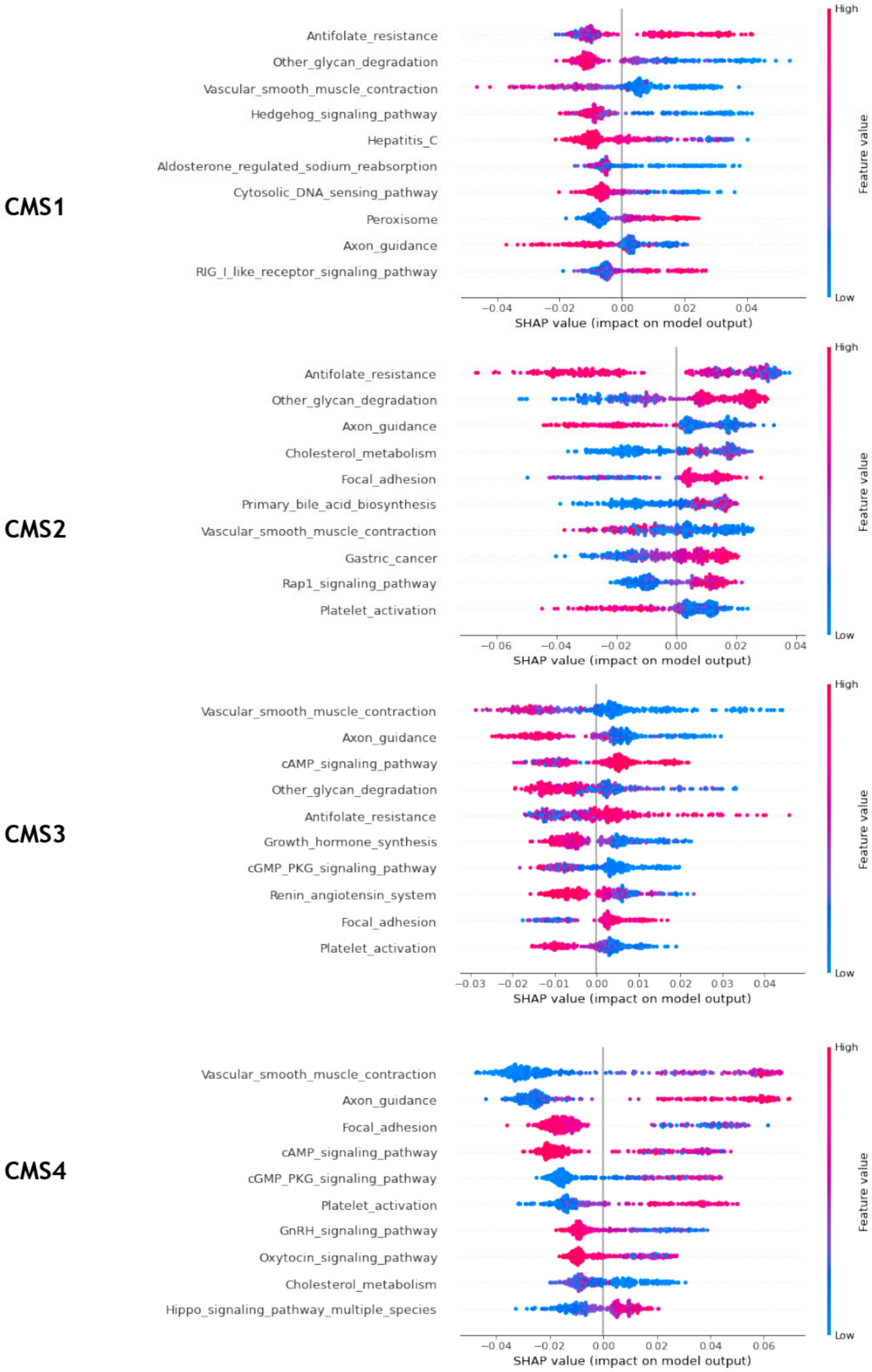

Figure S7. Top 10 pathways from SHAP for each subtype of COAD.
